# Poststroke Erectile Dysfunction in Cameroon: Prevalence, Associated Factors, and Quality of Life

**DOI:** 10.1155/2021/9988841

**Published:** 2021-12-03

**Authors:** Daniel Gams Massi, Gervais Ngoupayou Mountap, Hervé Edouard Moby, Frantz Guy Epoupa Ngalle, Sidick Mouliom, Jacques Doumbe, Njankouo Yacouba Mapoure

**Affiliations:** ^1^Douala General Hospital, Douala, Cameroon; ^2^Faculty of Health Sciences, University of Buea, Buea, Cameroon; ^3^Faculty of Medicine and Pharmaceutical Sciences, University of Douala, Douala, Cameroon; ^4^Faculty of Medicine and Biomedical Sciences, University of Yaoundé 1, Yaoundé, Cameroon

## Abstract

**Background:**

Stroke is a severe disease due to its morbidity-mortality. It is the first cause of acquired disability including erectile dysfunction (ED). The purpose of this study was to determine the prevalence of ED in stroke patients at the Douala General Hospital, to identify associated factors and to evaluate their quality of life.

**Materials and Methods:**

A cross-sectional study was conducted over a period of seven months from November 2016 to May 2017 on two groups of patients in neurology, cardiology, and endocrinology units of the Douala General Hospital (Cameroon): stroke patients (stroke+) and nonstroke patients (stroke-). We collected sociodemographic and clinical data using a preestablished questionnaire. Erectile function was assessed using International Index of Erectile Function (IIEF-5). Associated and predictive factors were determined using univariate and multivariate analyses. Results were significant for a *p* value < 0.05.

**Results:**

A total of 269 patients were included, among them 87 stroke+ (32.34%) and 182 stroke- (67.66%) (controlled group). The mean age was 56.37 ± 12.89 years and 57.18 ± 10.24 years of stroke+ and stroke-, respectively (*p* = 0.608). Prevalence of poststroke ED was 64.4% (OR = 3.41, 95% CI: 1.99-5.82, *p* < 0.001). The average time of occurrence of the poststroke ED was 5 ± 5.85 months. Diabetes and dyslipidemia were the predictive factors of occurrence of poststroke ED. Depression was found both in stroke+ with ED and stroke+ without ED with no difference (*p* = 0.131).

**Conclusion:**

About two-thirds of stroke patients developed ED. Diabetes and dyslipidemia were predictive factors of ED in stroke patients.

## 1. Introduction

Stroke is the second leading cause of death in the world with 6.7 million deaths in 2012 [1]. It is also the leading cause of long-term disability and the second leading cause of dementia worldwide [2–4]. In Sub-Saharan Africa (SSA), stroke is the leading cause of hospitalization in neurology departments [5]. In Cameroon, the overall mortality rate of stroke is 26.8% in hospitalization at Douala General Hospital (DGH) [6]. Poststroke sequelae are common; among them is erectile dysfunction (ED), which is still poorly documented in the world and particularly in Africa [7]. Penile erection is a neurovascular event characterized by the dilation of arteries that cause the corpora cavernosa and corpora spongiosum of the penis to fill with blood; concurrently, the ischiocavernosus and bulbospongiosus muscles compress the veins of the corpora cavernosa, which prevents the blood from exiting [8]. According to Shiri et al., ED is a frequently occurring disorder that increases in incidence strongly between 50 and 75 years of age. Diabetes is a major determinant of ED, but hypertension, heart disease, arthritis, and stroke increase the incidence of ED only marginally [9]. However, many studies actually found a high prevalence of ED among stroke patients [7, 10, 11]. Many relationships have been established between ED and cardiovascular pathologies including hypertension, myocardial infarction, and stroke [12]. Several patients suffering from chronic cardiovascular pathologies will experience a decrease in libido, frequency of sexual activity, and erectile dysfunction [13]. Due to sociocultural habits, complaining of sexual disturbances remains a taboo, especially among men. This makes difficult for the diagnosis and management of erectile dysfunction in patients with neurological disorders [14]. In order to contribute to a better management of stroke patients, we proposed to determine the prevalence of ED, associated factors, and impact on the quality of life in stroke patients.

## 2. Patients and Methods

This was a cross-sectional study conducted to determine the prevalence and impact of ED in stroke patients at the Douala General Hospital (DGH), the main reference hospital in Cameroon. The approval for the realization of this study was obtained from the ethics committee of the University of Douala (No. CEI-UDo/818/16/2017/T). The participants included in this study have previously given their informed consent after detailed explanations of the nature and aim of the study.

The study was conducted over a 6-month period from December 11^th^, 2016, to May 31^st^, 2017 in the DGH's neurology, cardiology, and endocrinology units. Two groups of participants were designed. Group 1 included any male patients followed up for stroke (stroke+) confirmed by brain imaging (CT scan or MRI) and followed up for at least 3 months. Group 2 included any male patient who never had stroke (stroke -), followed up in outpatient cardiology and/or endocrinology units. We excluded from this study the patient with neurological disorders prior to stroke, altered consciousness, or patients who did not give their consent. Group 1 patients were contacted by telephone using the hospital's registry, and an appointment was made between Monday and Friday from 9:00 am to 4:00 pm depending on their convenience. They were interviewed during their consultation by the investigator and the specialist. Patients with motor deficits (reduced mobility) had their appointments at home. Some information was completed by the patient's spouse and found in the patient's medical file. The sampling method was convenience nonprobability, meaning all patients meeting our inclusion criteria and who agreed to participate in the study were enrolled. We collected data on sociodemographic and clinical data ((1) age, (2) occupation, (3) residence, (4) marital status, (5) history of hypertension, diabetes, dyslipidemia, smoking, and alcoholism, and (6) body mass index). Cerebrovascular risk factors are defined in Table 1 [6]. Stroke's type and etiologies of stroke according to the Trial of ORG 10172 in Acute Stroke Treatment (TOAST) classification for ischemic stroke, Glasgow Coma Scale, NIHSS at admission, in-hospital complications, duration of poststroke follow-up, and functional prognosis using Barthel's scale were also included. Diagnosis of ED was based on the interview according to the score of the International Index of Erectile Function (IIEF-5). It is a widely used self-report questionnaire to evaluate male sexual function. It consists of 5 items each scored on an ordinary scale of 5 points for a total of 25. Thus, the ED was classified as not interpretable (score 1 to 4), severe (score 5 to 10), moderate (11 to 15), mild (16 to 20), and normal erectile function (21 to 25). To identify confounding factors such as depression (which is a major cause of ED) in stroke patients, we used the Beck Depression Inventory which is a self-assessment questionnaire evaluating severity of depression in patients. It consists of 21 items of 4 sentences corresponding to degrees of symptom. The range of the scale goes from 0 to 40. The higher the score, the more the subject is depressed: no depression (0 to 4), mild depression (4 to 7), moderate depression (8 to 15), and severe depression (16 or more). Finally, the quality of life of stroke patients was evaluated using the Stroke Specific Quality of Life Scale (SS-QOL). It consists of 49 items which assess areas of the patient's quality of life related to mobility, energy, upper extremity function, mood, work and productivity, language, self-care, social roles, family roles, vision, language, thinking, and personality. Each item is rated out of 5 for a total score of 245 which corresponds to an optimal quality of life. The quality of life was therefore classified as follows: very poor quality of life (SS − QOL < 50), poor quality of life (SS − QOL = [50 − 100]), moderate quality of life (SS − QOL = [101 − 150]), good quality of life (SS − QOL = [151 − 200]), and optimal quality of life (SS − QOL > 200).

Data was analyzed using the Statistical Package for the Social Sciences (SPSS) software version 20.0. Analysis was performed in two steps: we first compare stroke patients versus patients without stroke. Then, we compared stroke patients with ED (stroke ED+) versus stroke patients without ED (stroke ED-). Continuous variables were expressed as median (extremes) and mean with standard deviation (SD). The chi-square test allowed us to evaluate the association between the categorical variables, and the Student *t*-test was used to compare continuous variables. The statistical significance threshold was defined for *p* < 0.05. Associated and predictive factors were determined using logistic regression analysis.

## 3. Results

During the study period, 269 patients participated in the study, including 87 stroke+ and 182 stroke- patients. The prevalence of ED was 64.4% (56/87) in stroke+ and 35.6% (63/182) in stroke- (*p* < 0.001, OR = 3.41, 95%CI = [1.99 − 5.82]). The means ages (SD) were 57 (12.9) years for stroke+ and 56 (10.2) years for stroke-. The mean ages (SD) of onset of ED were 51 (9.1) years in stroke+ and 49 (11.8) years in stroke-. The mean time of ED's onset after stroke was 5 (5.85) months. Concerning the stroke+ population, the mean age (SD) of patients with ED (stroke ED+) was 59 (9.1) years versus 55 (11.79) years in patients without ED (stroke ED-). Details on sociodemographic data are found in Table 2.

**Figure 1:**
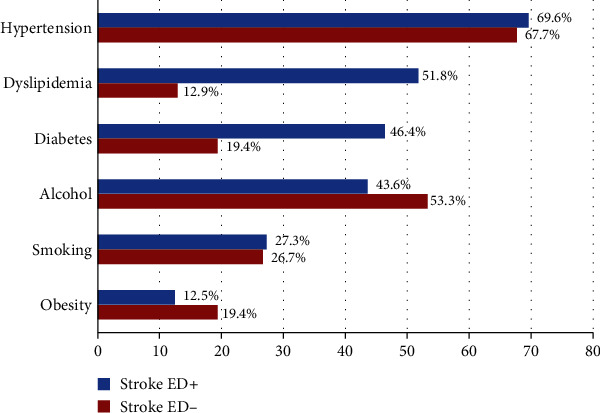
Cerebrovascular risk factors among stroke+ patients with and without ED.

Hypertension was the commonest cerebrovascular risk factor (CVRF) among stroke ED+ and stroke ED-. CVRFs in stroke population are represented in Figure 1. Diabetes (*p* = 0.012) and dyslipidemia (*p* < 0.001) were associated with ED in the univariate analysis.

Patients with right hemiplegia accounted for 51.8% of stroke ED+ patients versus 37% of stroke ED- patients (*p* = 0.115). Cerebral infarct was the main stroke type in both stroke ED+ and stroke ED- subgroups.  Table 3 shows comparison of clinical and paraclinical features between the stroke ED+ and stroke ED- populations.

Depression was found in 71.4% of stroke ED+ versus 54.8% of stroke ED- (*p* = 0.119). The mean SS-QOL was 145.3 ± 42.1 for stroke ED+ versus 174.7 ± 42.7 for stroke ED- patients (*p* = 0.003), meaning that the stroke ED+ group had a lower quality of life than the stroke ED- group.

Atherosclerosis was the most common etiology of ischemic stroke. In hemorrhagic stroke, hypertension was the main etiology in both patient groups (Table 4).

After multivariate analysis, diabetes and dyslipidemia appeared as predictors of poststroke ED, as shown in Table 5.

## 4. Discussion

This pioneer cross-sectional study in our setting is aimed at determining the prevalence of poststroke ED, associated and predictive factors, and its impact among male stroke patients. The prevalence of ED in poststroke patients in the DGH neurology unit is 64.4%. In Nigeria and Turkey, studies reported a hospital-based prevalence of 61.7% and 63.5%, respectively [7, 11]. Higher prevalence has been reported by Koehn et al. (78.9%) and Hilz et al. (82.14%) in Germany and the USA, respectively [15, 16].

More than two-thirds of our stroke ED+ patients had an ischemic stroke. Ischemic stroke has been reported to be the most frequent stroke type in our context [6]. However, ischemic stroke was not significantly associated with the occurrence of poststroke ED. Ossou-Nguiet et al. in Congo found that the type and topography of stroke did not seem to play any role in the occurrence of poststroke ED [17]. ED occurred between 3 months and 36 months of poststroke follow-up with an average of 5 ± 4.85 months. This delay is closed to the finding of Ossou-Nguiet et al. (4.7 ± 3.97 months) [17]. Within weeks to months following a stroke, patients, care-giver, and physicians mainly focus on the research of potential etiologies and intensive physiotherapy of motor deficit and may not be alerted on sexual function.

Cerebrovascular risk factors were more frequent in stroke ED+ versus stroke ED- participants, leading by hypertension on both populations. Several studies have reported hypertension and blood lowering medication as associated factor and predictor of ED among stroke patients [15, 18]. Hypertension is the most frequent risk factor of stroke [10, 19, 20]. However, in our study, we did not find a significant association between hypertension and poststroke ED.

Dyslipidemia, diabetes, right hemiplegia, and poor quality of life were the factors found to be associated with ED in stroke patients. Only dyslipidemia (*p* < 0.001) and diabetes (*p* = 0.012) were predictive factors for poststroke ED. Ossou-Nguiet et al. in Congo found hypercholesterolemia as the only predictive factor of poststroke ED (*p* = 0.007) [17]. Dyslipidemia has been reported several times as risk factor of erectile dysfunction among patients with and without stroke [15, 21]. Several hypotheses in the mechanisms by which dyslipidemia may lead to ED during stroke have been described. Dyslipidemia affects endothelial and smooth muscle cells of the penis, and oxidized LDL cholesterol contributes to the impaired relaxation of the corpus cavernosum [22].

Diabetes is known as a common cause of ED. Koehn et al. found diabetes as a comorbidity which is significantly associated with ED in stroke patients [15]. The proposed mechanisms of ED in diabetic patients include elevated end-products of glycation, increased levels of oxygen-free radicals, impaired nitric oxide (NO) synthesis, vascular endothelial changes, peripheral neuropathy, and psychological impact [23].

Using univariate analysis, right hemiplegia was significantly associated with poststroke ED. The study of Sikiru et al., on 105 poststroke patients grouped into left hemiplegic (*n* = 50), right hemiplegic (*n* = 55), and 40 age-matched control groups, found that stroke mostly affects erectile dysfunction in the right hemiplegic patients [24]. This same result has also been found in many literatures [25, 26]. The left hemispheric lesion may lead to a disruption of parasympathetic nervous system which is responsible for the regulation of erection [20]. Some studies did not find any association between the hemiplegia and the ED [19, 27]. Kimura et al. reported a link between left hemiplegia and the occurrence of ED in stroke [28]. Using voxel-based analysis, Winder et al. showed that ED after acute ischemic stroke was associated with lesions in the right occipital-parietal and thalamic areas integrating visual and somatosensory information, as well as lesions in the left insular and adjacent parietal-temporal areas contributing to generating and mapping visceral arousal states [29].

Our stroke patients with ED had lower quality of life than those without ED. This demonstrates the impact of ED on the quality of life after a stroke. Akinpelu et al. reported in Nigeria a significant association between poor quality of life and sexual dysfunction in stroke patients [7]. Sexual function is an essential part of quality of life in adults. However, ED in stroke patients is a common but underrecognized complication after stroke [30]. This is particularly true in our context where poststroke ED is underdiagnosed because of sociocultural considerations and taboos.

## 5. Conclusion

Two-thirds of stroke patients developed ED in this study. Diabetes and dyslipidemia were the predictive factors of the occurrence of poststroke ED. Erectile dysfunction remains underestimated among the stroke population. Careful history of patients and spouses may help to improve the early diagnosis and management of patients. Further study needs to be done to better understand the mechanism underlying poststroke erectile dysfunction.

## Figures and Tables

**Table 1:** Operational definition of cerebrovascular risk factors [6].

Cerebrovascular risk factors	Definition
Hypertension	Patient with medical history of hypertension, treated or not
Diabetes mellitus	Patient with medical history of diabetes, treated or not
Dyslipidemia	Patient with medical history of dyslipidemia, treated or not with one of these conditions
Alcohol consumption	Daily alcohol intake > 60 g/L
Obesity	2 methods were used to determine overweight or obesity: (i) if the body mass index is >30: obesity, BMI between 25 and 30 is overweight and (ii) when it is impossible to have the BMI, we used the abdominal circumference > 102 cm in male and >88 cm in female

**Table 2:** Sociodemographic characteristics of stroke+ populations.

	Stroke ED+ (%) (*N* = 56)	Stroke ED- (%) (*N* = 31)	OR (95% CI)	*p* value
Mean age ± SD	59 ± 9.1	55 ± 11.8		0.094
Age groups				
<45	4 (7.1)	7 (22.6)	0.3 (0.07–0.98)	0.049
45-60	29 (51.7)	14 (45.2)	1.3 (0.54–3.15)	0.656
>60	23 (41.1)	10 (32.3)	1.5 (0.58–3.68)	0.493
Marital status				
Married	46 (82.1)	26 (83.9)	0.9 (0.27–2.87)	0.999
Single	6 (10.7)	5 (16.1)	0.6 (0.17–2.24)	0.511
Widow	4 (7.1)	—	—	—
Worker				
Yes	50 (89.3)	28 (90.3)	0.89 (0.21–3.85)	0.999
No	6 (10.7)	3 (9.7)	1.12 (0.26–4.83)	0.999
Mode of payment				
Insurance	19 (33.9)	11 (35.5)	0.9 (0.37–2.34)	Ref
Individual	11 (19.6)	5 (16.1)	1.3 (0.39–4.06)	0.779
Individual+family	15 (26.8)	6 (19.4)	1.5 (0.52–4.44)	0.602
Family	10 (17.9)	8 (25.8)	0.6 (0.22–1.80)	0.416

**Table 3:** Clinical and paraclinical characteristics of stroke patients.

	Stroke ED+ (%) (*N* = 56)	Stroke ED- (%) (*N* = 31)	OR (95% CI)	*p* value
NHISS				
<15	23 (41.1)	12 (38.7)	1.1 (0.45–2.7)	0.999
≥15	33 (58.9)	19 (61.3)	0.9 (0.37–2.22)	
Systolic BP				
≤140	12 (21.4)	7 (22.6)	0.9 (0.33–2.69)	0.999
>140	44 (76.6)	24 (77.4)	1.1 (0.37–3.08)	
Blood sugar				
≤1.26	19 (33.9)	13 (41.9)	0.7 (0.30–1.75)	0.493
>1.26	37 (66.1)	18 (58.1)	1.4 (0.57–3.47)	
BMI				
18–25	23 (41.1)	18 (58.1)	0.5 (0.21–1.23)	0.179
25-30	27 (48.2)	7 (22.6)	3.2 (1.18–8.61)	0.023
>30	6 (10.7)	6 (19.4)	0.5 (0.15–1.71)	0.334
Hemiplegia/paresis				
Left	16 (28.6)	15 (48.4)	0.4 (0.17–1.06)	0.101
Right	29 (51.8)	10 (32.3)	2.3 (0.9–5.65)	0.115
Stroke type				
Ischemic	40 (71.4)	18 (58.1)	1.8 (0.72–4.53)	0.240
Hemorrhagic	16 (28.6)	13 (41.9)	0.6 (0.22–1.39)	

BMI: body mass index; BP: blood pressure; ND: neurological deficit; NIHSS: National Institutes of Health Stroke Scale.

**Table 4:** Etiologies of acute ischemic stroke and hemorrhagic stroke.

Etiologies	Stroke ED+ (%) (*N* = 32)	Stroke ED- (%) (*N* = 12)	OR (95% CI)	*p* value
Acute ischemic stroke				
Atherosclerosis	12 (37.5)	3 (25)	1.8 (0.41-7.99)	0.436
Cardioembolic	6 (18.8)	1 (8.3)	2.5 (0.27-23.64)	0.400
Small vessel disease	4 (12.5)	3 (25)	0.4 (0.8-2.29)	0.313
Arterial dissection	8 (25)	4 (33.3)	0.7 (0.16-2.82)	0.580
Other causes	1 (3.1)	1 (8.3)	0.4 (0.02-6.17)	0.460
Undetermined	8 (25)	4 (33.3)	1.1 (0.31–4.09)	0.999
Hemorrhagic stroke				
Hypertension	11 (78.6)	9 (69.2)	1.6 (0.29-9.26)	0.580
AVM	—	2 (16.7)	—	0.112
Other causes	3 (18.8)	2 (12.5)	1.5 (0.21-10.81)	0.686

**Table 5:** Predictive factors of poststroke ED.

	Stroke ED+ (%) (*N* = 56)	Stroke ED- (%) (*N* = 31)	aOR (95% CI)	*p* value
Diabetes mellitus	26 (46.4)	6 (19.4)	4.8 (0.98-23.11)	0.042
Dyslipidemia	29 (51.8)	4 (12.9)	4.7 (1.08-20.25)	0.039

aOR: adjusted OR.

## Data Availability

The data used to support the findings of this study are available from the corresponding author upon request.
